# Clinical and Preclinical Postgraduate Training in Endodontic Education: A Transnational Exploratory Survey

**DOI:** 10.1016/j.identj.2025.100861

**Published:** 2025-06-13

**Authors:** Venkateshbabu Nagendrababu, Vellore Kannan Gopinath, Mohannad Nassar, Naresh Shetty, Anil Kishen, Paul V. Abbott, Henry F. Duncan

**Affiliations:** a Department of Restorative Dentistry, University of Sharjah, College of Dental Medicine, Sharjah, United Arab Emirates; b Department of Orthodontics, Pediatric and Community Dentistry, University of Sharjah, College of Dental Medicine, Sharjah, United Arab Emirates; c Department of Oral and Maxillofacial Surgery, College of Dentistry, Texas A&M University, Dallas, Texas, USA; d Faculty of Dentistry, University of Toronto, Mount Sinai Health System, Mount Sinai Hospital, Toronto, Canada; e UWA Dental School, The University of Western Australia, Perth, Australia; f Division of Restorative Dentistry, Dublin Dental University Hospital, Trinity College Dublin, Dublin, Ireland

**Keywords:** Curriculum, Endodontics, Education, Postgraduate, Root canal, Survey

## Abstract

**Introduction and aims:**

Endodontic postgraduate programmes should offer students a wide range of experiences and training in all facets of the specialisation. This study aimed to assess the current postgraduate education practices in endodontics across dental schools worldwide.

**Methods:**

The current survey consisted of an online questionnaire that had been validated and piloted. It comprised 68 questions in three sections: general information about the programme, preclinical education, and clinical education. The survey included faculty members who teach postgraduate endodontics from one dental school in each participating country. The data was presented using simple descriptive statistics.

**Results:**

In total, 33 faculty members, with a response rate of 85%, from different countries completed the survey. The majority of dental schools that participated in the survey offer a 3-year postgraduate programme. The postgraduate students develop abilities in various instrumentation techniques, irrigation devices, sealers/cements, root canal filling techniques, surgery, and microscope use. Most of the schools conduct a final-year exit examination.

**Conclusions:**

Postgraduate education differs globally in its preclinical and clinical training. The survey offers initial insights into postgraduate endodontic education and can help establish mutual educational standards and key focus areas.

**Clinical relevance:**

The curricula for preclinical and clinical education in postgraduate endodontics programmes vary globally.

## Introduction

The structure of a postgraduate dental programme must provide specialised knowledge and develop skills beyond those acquired as an undergraduate student.^1^ Endodontic postgraduate programmes should provide students with a broad range of experiences and training in all aspects of the specialty. For programmes training endodontic specialists, the European Society of Endodontology (ESE) has described curriculum requirements that detail the aims and objectives, syllabus content, advanced training needs, and a multidisciplinary approach to managing endodontic conditions.^1^ The Australian Society of Endodontology (ASE) published the first set of educational guidelines for the training of endodontic specialists in Australia in 1979. These guidelines were subsequently revised several times by the Australian and New Zealand Academy of Endodontists (ANZAE). The most recent version was published in 2020, and they provide educational guidelines for the training of specialist endodontists in both Australia and New Zealand.^2^ Similarly, the American Association of Endodontists (AAE) offers general information and broadly outlines the necessary clinical experience for advanced endodontic programmes (https://www.aae.org/specialty/education-events/academics/advanced-programs-in-endodontics/).

In a 2018 survey of endodontic specialty training programmes in the United States of America (USA), 133 endodontic students reported satisfactory exposure to various procedures, products, and treatment protocols during their postgraduate training.^3^ On the contrary, a survey of Nigerian dentists highlighted that they believed their endodontic practice experience during postgraduate training had significant gaps in clinical teaching and practice.^4^ A survey of 3252 South African private dental practitioners highlighted the need for well-trained specialist endodontists.^5^ These findings emphasise the need for a potential review of the dental postgraduate curriculum to improve consistency in clinical decision-making among clinicians.

Undergraduate educational programmes in preclinical and clinical endodontic training have been evaluated in several countries, including the United Kingdom,^6^ Germany,^7^ and Spain.^8^ It is worth noting that similar studies regarding postgraduate endodontic education are needed. A bibliometric analysis comparing undergraduate and postgraduate endodontic education highlighted similarities and differences in their knowledge units and main topics.^9^ It was reported that, at undergraduate level, there was a strong focus on fundamental knowledge and teaching techniques. In contrast, at the postgraduate level, the emphasis was on advanced clinical knowledge and specialised techniques.^9^ Variations in postgraduate training within the same discipline negatively affect the clinical competence of future healthcare workers and also have implications for treatment quality and outcome. Thus, understanding global variations in postgraduate endodontic training has become crucial to ensure educational quality and facilitate benchmarking along with the adoption of clinical practice guidelines. Although there are data analysing undergraduate teaching in endodontics,^10^ there is a notable lack of clear data regarding clinical training standards and course content in postgraduate endodontic programmes across different regions.

To address this gap, the present study aimed to conduct a comprehensive international survey to evaluate the current status of preclinical and clinical postgraduate endodontic education in dental schools worldwide. Such a survey should assist in identifying gaps in knowledge and clinical skills, assess regional differences, and explorie the extent of adherence to national or international guidelines. This could contribute to the global improvement and potential standardisation of postgraduate curricula.

## Methods

### **The institutional research ethics committee (REC-24-02-27-01-F) approved the current survey**

The initial version of the questionnaire was developed based on the previous undergraduate/predoctoral education survey.^11^ It was subsequently modified and expanded to reflect the nature of postgraduate education. The initial version of the questionnaire was validated by six experts from various countries, each with at least 10 years of academic experience in the field of endodontics. The pilot study included five participants with a minimum of 3 years of academic experience in endodontics. These individuals were not included in the final survey. The validation and piloting processes are outlined in Supplementary Table 1. The final questionnaire (Supplementary Table 2) consisted of 68 questions with three sections (general information about the programmes, preclinical education, and clinical education).

Full-time or part-time faculty members from the department, division, or discipline of endodontics in one dental school from each country were eligible to participate in the current survey. The endodontic faculty member in each dental school was identified by contacting the dean or head of the school, the head of the discipline, or through personal contacts of the investigators of the current survey.

The survey investigators emailed potential participants, providing an essential project summary and requesting their consent to participate. Upon their acceptance, the investigators sent the faculty members the survey details and the link to the survey instrument. In the event that potential participants did not accept or did not respond, the principal investigator then communicated with another faculty member at the same institution or a different institution within the same country. The survey was initiated in September 2024 and closed in October 2024. The faculty members were informed that participating in the survey was entirely voluntary and that they could opt-out at any time. Faculty members were encouraged to complete the survey within the designated timeframe by sending them frequent messages via email and WhatsApp to increase the response rate. Participants who fail to complete the survey by the designated deadline, despite receiving reminder messages, are considered nonrespondents.

### Statistical analysis

The data were entered into an Excel spreadsheet (Microsoft Excel, Microsoft Corporation) and evaluated using SPSS software (Version 27, IBM Corp). Descriptive statistics were used to illustrate the data.

## Results

### Response rate

In total, 39 faculty members consented to participate in the survey, of which 33 responded and completed the questionnaire. Figure illustrates the 33 international universities from different countries that participated in the survey.

**Fig:**
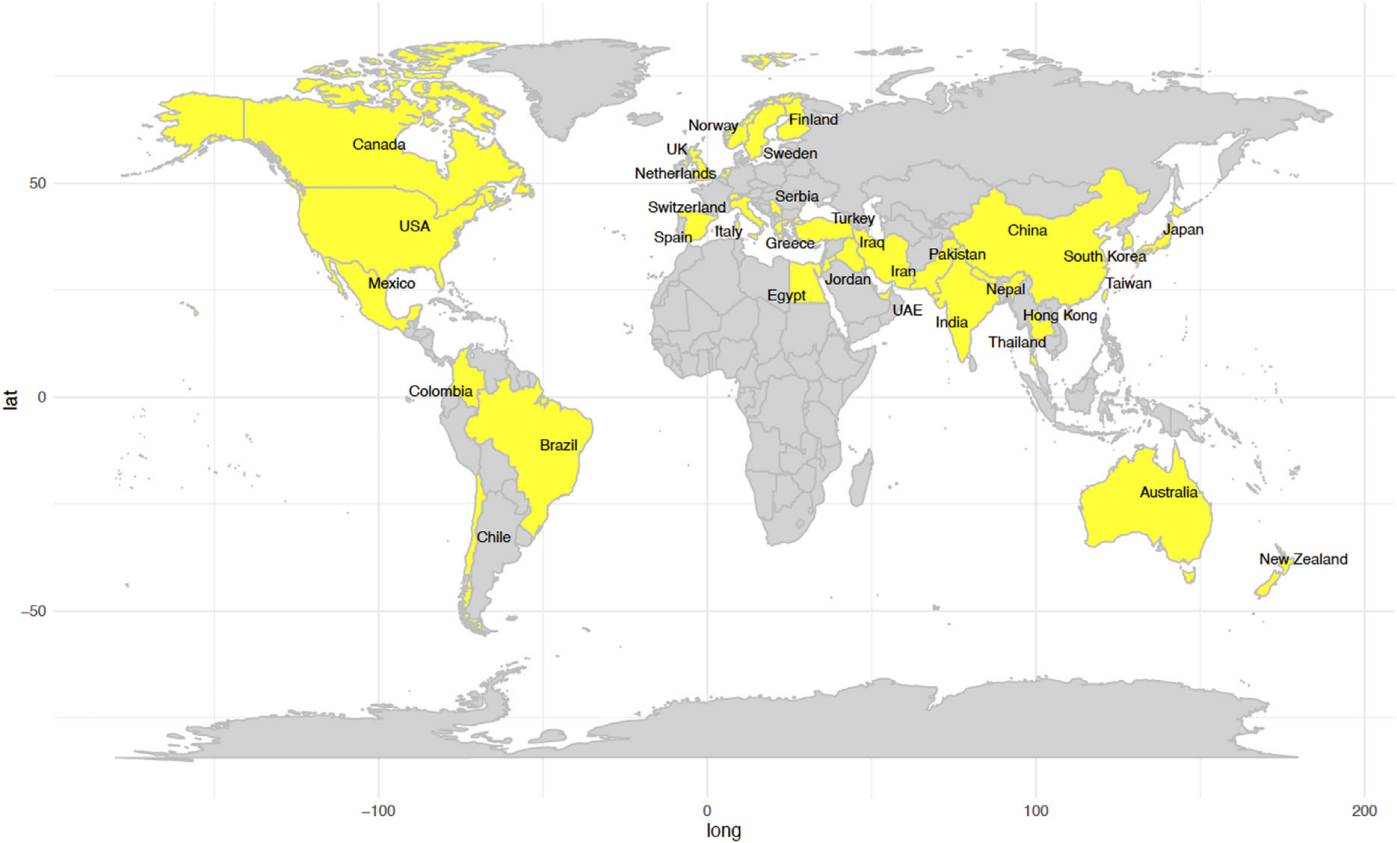
International universities from different countries participated in the survey highlighted in yellow.

### General information

A 3-year endodontic postgraduate programme was offered by 75.8% of the participating dental schools. Most dental schools required clinical experience and a high-Grade Point Average from a primary dental degree as prerequisites for admission to the postgraduate programme, while other factors such as clinical experience, interview, professional references, and practical tests were considered in some universities. International applicants are not accepted by 21% of the schools. The prerequisites for international and domestic applicants are not different in 42.4% of the schools. The processes used to select postgraduate students are presented in Supplementary Table 3. The number of students admitted to the course differed among the dental schools. The majority of the schools accepted students every year. The majority of schools also provided a full-time endodontic postgraduate programme. The teaching methods used in the postgraduate endodontic programmes are presented in Supplementary Table 4. In 69.7% of the schools, postgraduates taught undergraduate students in clinical or preclinical training for about 2 to 10 hours per week. A final-year exit examination was conducted in 84.8% of the schools, and most schools included a theory examination in addition to other assessment methods, such as a clinical portfolio, oral examination, and clinical examination. The postgraduate programme included a research component in 81.8% of the participating schools. The total percentage of time allocated for the research component was ‘not clear’ in 30% of the schools.

### Preclinical education

The supervising faculty members at 30.3% of the participating schools were only specialist endodontists. The ratio of faculty to students during preclinical training differed among the schools. The type of treatments students performed during preclinical training is presented in Supplementary Table 5. The number of hours dedicated to preclinical training varied among dental schools. The majority of the schools use natural teeth for preclinical training. Loupes and microscopes were used in 27.3% of the schools, whereas only microscopes were used in 33.3%. The stages of treatment where ultrasonic instruments are used in preclinical training are presented in Supplementary Table 6. The working length determination methods employed by 60.6% of the schools included apex locators and radiographs. The majority of schools use manual stainless-steel instruments and rotary file systems. The type of irrigating solutions used by the schools varies. The majority of schools used sodium hypochlorite. The irrigation techniques also varied with most schools using manual syringe irrigation with a needle (eg, side-vented needle). Most schools use cold lateral condensation/compaction with epoxy resin-based sealers as the root canal filling technique. In preclinical endodontic training, students must complete a minimum number of canals or teeth in 60.6% of the schools. The majority of the schools have both formative and summative assessments during the preclinical training. Most of the schools have no preclinical competence/objective practical test.

### Clinical education

Specialist endodontists are the only supervising faculty members during clinical training in 42.4% of the participating schools. The ratio of faculty to students during clinical training ranges from 1:1 to 1:8. Specialised training in cone beam computed tomography (CBCT) is provided in 48.5% of the schools. The presence of a CBCT image is mandatory for students before beginning nonsurgical RCT, retreatment, or following a traumatic dental injury in 24.2%, 36.4%, and 48.5% of the schools, respectively. The type of treatments students performed during clinical training is presented in Supplementary Table 7. The number of hours dedicated to clinical training varied among the schools. Most schools (81.8%) have dedicated postgraduate clinics for endodontics. The degree of case complexity of root canal treatments is determined by students in clinical training according to the AAE classification presented in Supplementary Table 8. Magnification is used for every case in 87.8% of the schools; 54.5% of the schools equip all dental chairs in postgraduate clinics with a microscope, while 30.3% of schools equip most chairs with a microscope. The stages of treatment where ultrasonic instruments are used in clinical training are presented in Supplementary Table 9. In clinical training, students are permitted to practice conservative endodontic access or minimally prepared canals in 60.6% of the schools. All schools employ radiographs and apex locators to determine working length. The types of root canal instruments used for canal shaping during clinical training are presented in Supplementary Table 10. All schools employ sodium hypochlorite and EDTA/EDTAC for irrigation. The chemical removal of the smear layer is practised in all schools. The final rinse protocol varied among schools regarding the type of irrigating solution, quantity, concentration, and time employed. The concentration(s) of sodium hypochlorite solution varied amongst the schools. Calcium hydroxide was only employed as an interappointment medicament (dressing) in 51.5% of the schools. The interappointment medicament placement technique used is presented in Supplementary Table 11. The protocol used to remove interappointment medicaments varied amongst the schools. Most schools utilise manual syringe irrigation with a needle (eg, a side-vented needle). Most schools use calcium silicate-based sealer/cement, cold lateral condensation/compaction, and single cone gutta-percha. A provisional coronal restoration was placed immediately after root canal treatment completion in 75.8% of the schools. Postgraduate endodontic students were typically involved in fabricating the definitive restoration in 51.5% of the schools. The students in postgraduate endodontics programme can perform the placement of posts in 60.6% of the schools. Students are taught and instructed to administer intravenous sedation and conscious sedation at only one and four of the participating schools, respectively. A minimum number of procedures that students are required to complete during their clinical training before graduation is set by only 42.4% of the schools. Summative assessments are only conducted during clinical training in 69.7% of the schools. There is no clinical competence/objective practical test in clinical endodontics in 63.6% of the schools.

## Discussion

To the best of the authors’ knowledge, the present survey is the first to assess postgraduate endodontic education in dental schools worldwide. The faculty of each selected dental school responded to an online questionnaire for this survey. Research indicates that questionnaires are advantageous for acquiring educational data.^7^^,^^8^^,^^11^ The current survey results indicate that postgraduate education varied in terms of preclinical and clinical training. This could be due to different curriculum guidelines in each country and different clinical operating models for the various schools. Interestingly, undergraduate endodontic education also shows considerable variation.^11^

It is clear that the majority of the participating schools provide a 3-year full-time endodontic postgraduate programme. The ANZAE, ASE, and ESE have recommended that the postgraduate programme be at least 3 years of full-time education.^1^^,^^2^ However, students can register on a half-time basis, provided that the educational experiences, including clinical experience and responsibilities, are equivalent to those of full-time students.^1^ In the current survey, prerequisites for admission at most schools necessitate clinical experience and a Grade Point Average from a primary dental degree, while other factors such as clinical experience, interview, references, and practical tests were considered in some universities. Furthermore, each school implements various parameters. Each school can determine its own prerequisites. Nevertheless, it is advisable to establish standardised, predetermined selection criteria to guarantee the highest quality of applicants. It is noteworthy that the ANZAE, ASE, and ESE recommend that schools only accept applicants who have at least 2 years of general dentistry experience.^1^^,^^2^ In 2014, a survey of programme directors in the USA regarding the selection of applicants for endodontic specialty programmes revealed that interview ratings, dental school class rank, and general practice residency or advanced education in general dentistry experience were significant factors when assessing applicants.^12^

The current survey revealed that only specialist endodontists were involved in postgraduate teaching in certain schools. Undergraduate endodontics teaching followed a similar pattern.^11^ A well-qualified faculty is the main criterion for an acceptable advanced education training programme.^1^ Faculty should be on the national endodontic specialist list in those countries where such a list exists.^1^^,^^2^ The current survey shows that preclinical and clinical faculty-to-student ratios vary greatly. The ESE recommends a faculty-to-student ratio of 1:4 for clinical training.^1^ It is recognised that the faculty-to-student ratio influences the capacity of supervising personnel to recognise students’ deficiencies and shortcomings.^13^

This survey also highlights that clinical training in all dental schools included vital pulp treatment (VPT). However, VPT is only taught in a limited number of schools during preclinical training. In the past, nonsurgical root canal treatment has been recommended for managing teeth diagnosed as irreversible pulpitis; however, recently VPTs have also been indicated for the successful management of teeth with this diagnosis.^14^ Given this shift, it would be more appropriate to integrate VPT into the preclinical curriculum. Furthermore, this survey emphasises the importance of conducting further research to gain a better understanding of the VPT education element in undergraduate curricula in both preclinical and clinical settings.

The majority of the dental schools participating in the current survey have a research component in their curriculum. The ESE recommends that students undertake a research project and present their findings in a formal thesis.^1^ According to the ANZAE guidelines, students must complete a research project that meets the individual university’s specifications as well as the requirements of the Australian Qualifications Framework Level 9 Extended or New Zealand Qualifications Framework Level 10. These Frameworks are set by the respective government educational authorities in Australia and New Zealand. Students are encouraged to present the findings of their research at conferences and publish them in dental journals.^2^ Postgraduate students are required to teach undergraduate students, provided they adhere to the appropriate educational framework. The ESE recommends restricting clinical teaching for undergraduate students to no more than 10% of the overall clinical activity. Evidence of peer supervision and quality control should accompany such teaching activities.^1^

The use of microscopes is taught at the graduate level in all Commission on Dental Accreditation-approved endodontic specialty programmes in the USA.^15^ Similarly, the ESE guidelines recommend that students should have a thorough understanding of microscopes and be able to apply their clinical skills effectively.^1^ However, obstacles such as inadequate faculty training, costs, and clinical space must be resolved to encourage more extensive use of microscopes. The ESE^16^ and the Joint AAE/American Academy of Oral and Maxillofacial Radiology^17^ Position Statement outlined that CBCT is a technology that has revolutionised the way patients undergo endodontic treatment. In 2018, Rabiee et al,^18^ investigated whether the endodontic residency programmes in the USA effectively equip their graduates with the skills for optimal use of CBCT. The study revealed that despite the significant variations in the teaching of CBCT at both classroom and clinical settings among the programmes, there was an agreement on the importance of establishing proficiency in the use of CBCT for future endodontists.^18^ Given this significance, it would be more advantageous to incorporate judicious CBCT training into the postgraduate endodontic curriculum.

The preclinical teaching of endodontic surgery at selected schools indicates a growing focus on this treatment approach. However, there appears to be a need for advanced and practical models that can provide realistic simulations of the procedure to enhance its integration into the preclinical curriculum.^19^ Nevertheless, most of the participating schools also offer clinical training in this procedure, and this substantiates the notion that it is an integral component of endodontic treatment. In addition, with the anticipation of increased demand for periapical surgery, graduate students and future endodontists will be expected to receive adequate training to incorporate this skill into their practice.^20^ The incorporation of preclinical training in managing traumatic dental injuries in only few schools may indicate a lack of effective simulation-based education in this area. Interestingly, two decades ago, bovine deciduous incisors were utilised to instruct obturation techniques in open apex teeth.^21^ However, recent models have been developed for the management of traumatic dental injuries in undergraduate education,^22^^,^^23^ suggesting a need for more sophisticated models that meet the educational requirements at a postgraduate level.

Administering local anaesthesia, sedation, and general anaesthesia is an essential component of dental practice, as recommended by the American Dental Association.^24^ Intravenous sedation is a technique that reduces fear and anxiety during dental treatment.^25^ Fear and anxiety may present hurdles for certain patients who are undergoing endodontic treatment. The anxiety can be directly linked to discomfort experienced during endodontic treatment.^26^ Therefore, sedation may be advantageous for certain patients^27^ and there is high demand for it in endodontics.^28^ A survey was conducted among 31 endodontic programme directors in the USA, and 13 reported that they taught IV sedation.^29^ However, administering IV sedation is rarely considered within the scope of endodontic postgraduate students. Nevertheless, the inclusion of IV sedation into the postgraduate curriculum will enhance the confidence of postgraduates in administering IV sedation in daily practice, thereby benefiting nervous or uncooperative patients. Despite this, there may be local regulatory constraints that inhibit this approach in both the teaching and the practice of endodontics. This is because some regulators require that a separate operator (apart from the clinician providing the dental/endodontic treatment) must administer the sedative drugs and monitor the patient during treatment, as well as during the recovery of the patient. The operator administering the sedation must be qualified either in dentistry or medicine and requires specific training and formal qualifications in sedation techniques.

The current survey results also indicate that postgraduate students acquire expertise in a diverse array of instrumentation techniques, irrigation devices, sealers/cements, and root canal filling techniques, contributing to the overall breadth of their training experience. It is noteworthy that the protocols for final irrigation techniques vary among the participating schools in terms of the type of irrigating solution employed, volume of irrigant, concentration of irrigant, and duration. Although irrigant type, order, and concentration were recently described by the ESE in their clinical practice guideline,^30^ the reported diversity may be attributed to the absence of robust literature in this area and the lack of an accepted standardised protocol. For example, currently, there is no clear guidance on the time or quantity of irrigant solution required during root canal treatment. However, all participating dental schools employ chemical methods for smear layer removal. In 2021, Tsotsis et al^31^ examined this issue in a recent survey of AAE-endodontist members, revealing that recent graduates are more likely to practice smear layer removal than those who graduated 10 to 25 years ago. Both the literature and educational practices tend towards supporting its removal, suggesting that the benefits outweigh any potential risks.^32^ A notable finding of this study is that the vast majority of schools reported using syringes or spiral fillers to place interappointment medication. The preference for these methods over manual files is likely linked to the goal of ensuring effective medication placement. However, concerns have been raised regarding the risks and dangers associated with syringe delivery techniques, particularly in teeth close to anatomical structures such as the inferior dental nerve.^33^

The ANZAE guidelines have recommended students spend time observing and helping endodontists in private or public clinics. This experience provides a variety of benefits, including the development of skills in practice administration, writing reports, and ethics.^2^ The ESE recommended that students treat at least 180 patients during their training period with a minimum distribution of 60 patients annually. These suggested numbers are not strict benchmarks but rather the minimum average number of cases required to graduate as a specialist with the necessary expertise and skills.^1^ Specifying a predefined minimum number of cases, which each school can decide based on the infrastructure and number of patients reporting to the clinic, may be advantageous. However, other views suggest that quality, not quantity, may be more critical during training. At the end of the programme, there must be a final examination conducted by a group of examiners, which should include at least one external examiner who is appropriately qualified.^1^ The ANZAE guidelines suggest that at least one of the external examiners should be from a different state within Australia or New Zealand.^2^

The current survey received a response rate of 85%, which could be considered strength of the survey. However, the survey has the following limitations: (1) This survey was limited to a single dental school per country, which may not provide a comprehensive representation of all dental schools within that country. Therefore, future efforts could involve conducting a thorough survey across multiple schools within the same country to assess standardisation. (2) Although the authors intended to distribute the survey worldwide, several nations did not participate. Some of these were due to the absence of any formal postgraduate training in those countries (e.g., Ireland, Denmark). (3) The project investigators selected the participants through their personal contacts, which may have introduced a degree of selection bias. (4) As a result of feedback from participating schools, in order to maintain curriculum confidentiality, the identity of each university is not disclosed in this report. The curriculum will differ from university to university, but the number of countries included in the current survey is sufficient to accommodate geographical differences. Countries not involved in the current survey will be urged to participate in a similar survey in the future. (5) The information provided by a single faculty member in the dental school was the primary focus of the current survey. In the future, a survey can be conducted with the participation of the programme lead or the head of the discipline to obtain more accurate and authenticated information about the curriculum.

## Conclusion

Postgraduate education in endodontics varies among the dental schools included in the survey. The majority of participating dental schools offered a 3-year endodontic postgraduate programme. Postgraduates taught undergraduate students in clinical or preclinical training in 69.7% of the schools. In 84.8% of the schools, a final-year exit examination was conducted. This current survey offers valuable initial insights into the current state of postgraduate education in endodontics across different regions. It may help establish mutually beneficial educational standards and promote discussions about the existing differences in the programmes.

## Conflict of interest

The authors have stated explicitly that there are no conflicts of interest in connection with this article.

## Appendix Supplementary materials

Image, application 1

## Footnotes

Supplementary material associated with this article can be found in the online version at doi:10.1016/j.identj.2025.100861.
