# Supplementary material for: Clinical and Preclinical Postgraduate Training in Endodontic Education: A Transnational Exploratory Survey
Source: Int Dent J. 2025 Jun 13;75(4):100861. doi: 10.1016/j.identj.2025.100861 (PMC12205319; doi:10.1016/j.identj.2025.100861)
Supplement: Supplementary file 1 [file mmc1.docx]

**Supplementary Tables**

| **Supplementary Table 1 – Validation and piloting process**  **Validation^1^**^,2,3^  Experts assessed items for relevance, clarity, simplicity, and ambiguity using a four-point Likert scale. For this survey, the I-CVI, which measures item content validity, and the S-CVI/Ave, which measures scale content validity, were used. The content validity of each question was determined by dividing the sum of experts' 3 or 4 ratings (indicating agreement) by the total number of experts. S-CVI /Ave was derived by averaging I-CVI. 0.83 is the minimum S-CVI. Experts also provided feedback on survey item sequencing, grammar, and the need for new or deleted items. After the first round, validation was complete. The I-CVI for each item in the survey's four domains was over 0.83, and the S-CVI/Ave values for relevance, clarity, simplicity, and ambiguity were 0.94, 0.97, 0.96, and 0.96. Based on the I-CVI and S-CVI/Ave scores, the questionnaire scale exhibited sufficient content validity.  **Piloting**  The electronic version of the questionnaire was developed by the project leader (VN) using Google Docs after the process of validation was completed.   The participants were also allowed to express their opinions on the item flow of the questionnaire, the ease of question comprehension, and to assign scores to each item in the survey. All participants deemed the questionnaire's item flow logical and simple to understand. They also had the opportunity to express any additional perspectives or provide unrestricted commentary on the survey.  **References**   1. Polit, D.F., Beck, C.T. (2006) The content validity index: are you sure you know what’s being reported? Critique and recommendations. *Research in Nursing & Health,* 29, 489–97. 2. Polit, D.F., Beck. C.T., Owen, S.V. (2007) Is the CVI an acceptable indicator of content validity? Appraisal and recommendations. *Research in Nursing & Health*, 30, 459–67. 3. Yusoff MSB (2019) ABC of content validation and content validity index calculation. Education in Medicine Journal 11: 49–54. |
| --- |

**Supplementary Table 2: Questionnaire**

| **SECTION A – General information about the program (entry requirements, teaching methods, exit exam)**   1. In which country do you presently work as teaching faculty at a post-graduate program in Endodontics? 2. What is the length of the endodontic postgraduate programme at the dental school where you are presently a faculty member? (If needed, you can select more than one answer). (*1-4 year, others*). 3. Are there any specific prerequisites for applicants who are applying for acceptance into the postgraduate programme in the discipline of Endodontics at your dental school? (If needed, you can select more than one answer). *(Clinical experience, Academic experience, Grade (GPA) from primary degree, Postgraduate qualifications (e.g., diploma, masters), Country of undergraduate qualification, External organisation exams (e.g., Royal College Fellowship), Others).* 4. Are the prerequisites for international applicants different to those for domestic applicants (e.g., exam scores, language tests)? *(Yes/No, We don’t accept international students)**.* 5. If YES, for question 4, specify the differences between national and international applicants with respect to prerequisites. If "No" for question 4, mention "NA". 6. What is the process used to select postgraduate students in the discipline of endodontics at your dental school? *(If needed, you can select more than one answer) (Theory exam, Preclinical exam, Clinical exam, Interview, Presentation, External organisation exams, Grade point average (GPA), Achievements, Recommendation/supporting letter, Research potential, Clinical experience, Academic experience, Others).* 7. How many students do you admit into the 1^st^ year of your course? 8. Do you accept students every year or at other time intervals? 9. What type of endodontic postgraduate program is offered in your dental school? (If needed, you can select more than one answer) *(Full-time, Part-time, Distance learning, Hybrid course, Others).* 10. What teaching methods are used in postgraduate endodontic program? *(If needed, you can select more than one answer) (Face-to-face lectures, Seminars and tutorials, Independent study, Assignment/Projects, E-learning, Laboratory & practical learning, Video, Manuals, Reading lists, Clinical cases, Problem based learning, Chairside teaching, Reflective portfolio, Other).* 11. Do postgraduate students teach undergraduate students in clinical or preclinical training? *(Yes/No)*. 12. How many hours per week, on average, do postgraduate students teach undergraduates in clinical or preclinical teaching? (If “Yes”, for question 11, please provide the average number of hours; if “No” for question 11, please mention "0.") 13. Do you have a final year exit examination in your dental school? (Yes/NO) 14. What type(s) of assessments are conducted in the final year exit exam for endodontics? (If needed, you can select more than one answer) (If No for question 13, please select “Others” and mention "0.") *(Theory exam (Written), Oral exam, Clinical exam, Assessment of clinical portfolio, Competence (Lab), Competence (Clinic), Others).* 15. Is there a research component in the endodontic postgraduate programme at your dental school? *(Yes/No).* 16. What is the approximate total percentage of time throughout the endodontic postgraduate programme that is allocated for the research component of the course? (If Question 15 is answered YES, please provide the percentage; If the question 15 is answered NO, please indicate "0."; if the percentage is unclear or you don’t know exactly, please indicate "Not clear.").   **Section B – Preclinical education**   1. What is the status of the supervising faculty member during pre-clinical endodontic training? (If needed, you can select more than one answer) *(Consultant/ Faculty in Restorative Dentistry, Specialist Endodontist, General Dental Practitioner, General Dental Practitioner with special interest in Endodontics, Senior graduate/postgraduate students, Other).* 2. What is the faculty: student ratio during pre-clinical endodontic training? 3. What type of treatments do students perform during pre-clinical endodontic training? (If needed, you can select more than one answer) *(Vital pulp therapies including pulp capping and pulpotomy, Root canal treatment of single-rooted teeth, Root canal treatment of multi-rooted teeth, Root canal re-treatment, Endodontic surgery, Treatment of teeth with open apices, Management of traumatic dental injuries, Perforation repair, Management of tooth resorption, Removal of fractured instruments, Other).* 4. How many hours are dedicated to pre-clinical endodontic training? 5. What types of root canal models are used during pre-clinical endodontic training? (If needed, you can select more than one answer) *(Canals in natural teeth, Canals in plastic teeth available commercially, Canals in 3D printed teeth, Canals in acrylic blocks with simple curves, Canals in acrylic blocks with S-shaped curves, Other).* 6. What type of magnification is used during pre-clinical endodontic training? (If needed, you can select more than one answer) *(Not used, Loupes, Microscope, Other).* 7. During which stage of treatment are ultrasonic instruments used in pre-clinical endodontic training? *(If needed, you can select more than one answer) (Not used, Access cavity preparation/refinement , Troughing, Irrigant agitation, Retrieval of fractured instrument, Post removal, Space preparation for retrograde filling, Other).* 8. What method(s) of working length determination is/are used during pre-clinical endodontic training? (If needed, you can select more than one answer) *(Radiographs, Electronic apex locator, Both radiographs and apex locator, Other)*. 9. What root canal instruments are used for canal shaping during pre-clinical endodontic training? *(If needed, you can select more than one answer) (Manual stainless instruments, Manual Ni-Ti files, Rotary systems, Reciprocating systems, Other).* 10. Which irrigating solutions are used during pre-clinical endodontic training? (If needed, you can select more than one answer) *(None, Water, Saline, Local anaesthetic solution, Sodium hypochlorite, Chlorhexidine, EDTA/EDTAC, Other).* 11. Which type of irrigation activation/agitation methods and devices are used during pre-clinical endodontic training? (If needed, you can select more than one answer) *(Manual: Syringe irrigation with needle (e.g. side-vented needle), Manual Brushes (e.g. Endobrush), Manual activation with gutta percha, Machine assisted, rotary brush, Machine assisted, sonic (e.g. Endo Activator), Machine assisted, ultrasonic, Machine assisted, pressure alternation devices (e.g. EndoVac), Others, Don’t use any additional irrigation activation/agitation methods).* 12. Which type of sealers/cements are used during pre-clinical endodontic training? (If needed, you can select more than one answer) *(Zinc oxide-based sealer/cement, Calcium hydroxide-based sealer/cement, Resin-based sealer/cement, Calcium silicate-based sealer/cement, Others).* 13. Which method of root canal filling is used during pre-clinical endodontic training? (If needed, you can select more than one answer) *(Matched single cone gutta-percha, Cold lateral condensation/compaction, Warm vertical condensation/compaction, Thermoplastic injection technique, Carrier-based gutta-percha, Paste fillers, Other).* 14. Is there a minimum number of canals or teeth that students are required to complete in pre-clinical endodontic training? (Some University requirements are based on number of “canals” rather than “teeth”. Hence, we mentioned “number of canals or teeth”. Based on your curriculum - you can select the number of canals or teeth” in Question 31) *(Yes (If yes, please provide details in Question 31), No).* 15. What is the minimum number of canals or teeth that students required to complete in pre-clinical endodontic training? 16. What type of assessments are carried out during pre-clinical endodontic training? (If needed, you can select more than one answer) *(Formative, Summative, Others).* 17. Do you have a preclinical competence/ objective practical test? *(Single root, Multi root, Vital pulp treatment, Other, None).*   **Section C – Clinical education**   1. What is the status of the supervising faculty member during clinical endodontic training? (If needed, you can select more than one answer) *(Consultant / Faculty in Restorative Dentistry, Specialist Endodontist, General Dental Practitioner, General Dental Practitioner with special interest in Endodontics, Other).* 2. What is the faculty: students’ ratio during clinical endodontic training? 3. Do students acquire special training (e.g., level 1/ level 2) needed to justify, carry out and interpret a Cone beam computed tomography (CBCT) examination? (Yes/No). 4. Within your program, is CBCT imaging typically required before students begin non-surgical root canal treatment procedures? (Yes/No). 5. Within your program, is CBCT imaging typically required before students begin retreatment procedures? *(Yes/No).* 6. Within your program, is CBCT imaging typically required before students commence endodontic treatment of a tooth following a traumatic dental injury? *(Yes/No).* 7. What types of endodontic treatments do students perform during clinical endodontic training? (If needed, you can select more than one answer) *(Vital pulp therapies including pulp capping and pulpotomy, Root canal treatment of single-rooted teeth, Root canal treatment of multi-rooted teeth, Root canal re-treatment, Endodontic surgery, Treatment of teeth with open apices, Management of traumatic dental injuries, Perforation repair, Management of tooth resorption, Internal bleaching, Removal of fractured instruments, Other).* 8. How many hours are dedicated to clinical endodontic training in the entire program (total for all years)? 9. Does your school have a dedicated postgraduate clinic for endodontics? (Yes/No). 10. What degree of case complexity of root canal treatments is performed by students in clinical endodontic training according to the American Association of Endodontists (AAE) classification? (If needed, you can select more than one answer). *(Simple (low difficulty), Moderate (moderate difficulty), Complicated (high difficulty)).* 11. What type of magnification is used during clinical endodontic training? (If needed, you can select more than one answer) *(Not used, Loupes, Microscope, Other).* 12. Are the dental chairs in your postgraduate clinic equipped with a microscope? *(Yes/No/Most).* 13. During which stage of treatment are ultrasonic instruments used in clinical endodontic training? (If needed, you can select more than one answer). *(Not used, Access cavity preparation/refinement, Troughing, Irrigant agitation, Retrieval of fractured instrument, Post removal, Space preparation for retrograde filling, Other).* 14. Are students allowed to practice conservative endodontic access or minimally prepared canals in clinical training? (Yes/No). 15. What method of working length determination is used during clinical endodontic training? (If needed, you can select more than one answer). *(Radiographs, Electronic apex locator, Both radiographs and electronic apex locator, Other).*   49. Which root canal instruments are used for canal shaping during clinical endodontic training? (If needed, you can select more than one answer). *(Manual stainless instruments, Manual Ni-Ti files, Rotary systems, Reciprocating systems, Other).*   1. Which irrigating solutions are used during clinical endodontic training? (If needed, you can select more than one answer). *(None, Water, Saline, Local anaesthetic solution, Sodium hypochlorite, Chlorhexidine, EDTA/EDTAC, Other).* 2. Is smear layer removal practiced clinically by the postgraduate students? *(Yes/No).* 3. What final rinse protocol (irrigating solution type, quantity (ml), concentration (%), time (minutes) is recommended within the program? E.g., 10 ml 3% NaOCl for 5 minutes, followed by 2 ml 17% EDTA for 5 min and then saline. 4. Which concentration(s) of sodium hypochlorite solution is/are used in clinical endodontic training? (If needed, you can select more than one answer). *(O.5 %, 1 % ,2.5 %, 3 %, 4 %, 5.25 %, Other).* 5. Which type of irrigation activation/agitation methods and devices is used in clinical endodontic training? (If needed, you can select more than one answer). *(Manual: Syringe irrigation with needle (e.g., side-vented needle), Manual Brushes (e.g. Endobrush), Manual activation with gutta percha, Machine assisted, rotary brush, Machine assisted, sonic (e.g., EndoActivator), Machine assisted, Ultrasonic*   *Machine assisted, Pressure alternation devices (e.g., EndoVac), Others, Don’t use any additional irrigation activation/agitation methods).*   1. Which type of sealers/cements are used in clinical endodontic training? (If needed, you can select more than one answer). (*Zinc oxide-based sealer/cement, Calcium hydroxide-based sealer/cement, Resin-based sealer/cement, Calcium silicate-based sealer/cement, Others).* 2. Which method of root canal filling is used in clinical endodontic training? (If needed, you can select more than one answer). *(Single cone gutta-percha, Cold lateral condensation/compaction, Warm vertical condensation/compaction, Thermoplastic injection technique, Carrier-based gutta-percha, Paste fillers, Depends on the requirements of the individual case, Other).* 3. Which types of inter-visit medicament (dressing) are used in clinical endodontic training? (If needed, you can select more than one answer). *(No medicament – canals left empty in between appointments, No medicament – single visit treatment whenever possible, Calcium hydroxide, Corticosteroid: antibiotic paste, Antibiotics, Other).* 4. What is the most commonly used interappointment medicament placement technique within the clinics?. (*Manual files, Spiral filler, Direct syringe injection, Others).* 5. What is the most commonly used protocol to remove interappointment medicament from root canals? 6. What type of restoration is placed immediately after completion of root canal treatment in clinical endodontic training? (Provisional restoration, Definitive restoration) 7. Are the endodontic postgraduate students typically involved in fabrication of the definitive restoration? *(Yes/No).* 8. When a case needs a post placed before the fabrication of the definitive restoration, is post placement required to be done by the endodontic program students? *(Yes/No).* 9. Are the students clinically trained to administer IV-sedation? *(Yes/No).* 10. Are the students clinically trained to administer nitrous oxide conscious sedation? *(Yes/No).* 11. Is there a minimum number of procedures that students are required to complete in clinical endodontic training before graduation? *(Yes (If yes, please provide details in Question 66). /No).* 12. What is the minimum number of canals or teeth that students required to complete in clinical endodontic training? 13. What type(s) of assessments are conducted during the clinical endodontic years? (If needed, you can select more than one answer). *(Formative, Summative, Others).* 14. Do you have a clinical competence/ objective practical test in endodontics? *(Single root, Multi root, Vital pulp treatment, Other, None*). |
| --- |

**Supplementary Table 3: Process used to select postgraduate students**

| **Options** | **Percentage (%)** |
| --- | --- |
| External organization exams; Grade point average (GPA); Clinical experience | 3.0 |
| Grade point average (GPA) | 3.0 |
| Interview | 3.0 |
| Interview; Achievements; Clinical experience; Academic experience | 6.1 |
| Interview; Achievements; Recommendation/supporting letter; Clinical experience | 6.1 |
| Interview; Achievements; Recommendation/supporting letter; Clinical experience; Academic experience | 3.0 |
| Interview; Grade point average (GPA); Achievements; Recommendation/supporting letter; Research potential; Clinical experience | 3.0 |
| Interview; Grade point average (GPA); Achievements; Recommendation/supporting letter; Research potential; Clinical experience; Academic experience | 6.1 |
| Interview; Grade point average (GPA); Achievements; Research potential; Clinical experience; Written exams | 3.0 |
| Interview; Grade point average (GPA); Research potential; Clinical experience; Academic experience | 3.0 |
| Interview; Presentation; Achievements; Recommendation/supporting letter; Research potential; Clinical experience; Academic experience; FINAL GRADUATION GRADE - PUBLICATIONS | 3.0 |
| Interview; Presentation; Achievements; Research potential; Clinical experience; Academic experience | 3.0 |
| Preclinical exam; Interview; Grade point average (GPA); Achievements; Multiple Mini Interviews | 3.0 |
| Recommendation/supporting letter; Research potential; Clinical experience; Academic experience; proposal of applicants (master program + specialist training; but no certificate of specialist) | 3.0 |
| Theory exam | 3.0 |
| Theory exam; Clinical exam; Interview; External organisation exams | 3.0 |
| Theory exam; Clinical exam; Interview; Grade point average (GPA); Recommendation /supporting letter; Research potential; Academic experience | 3.0 |
| Theory exam; Clinical exam; Presentation; Grade point average (GPA); Recommendation /supporting letter | 3.0 |
| Theory exam; External organisation exams; Grade point average (GPA) | 3.0 |
| Theory exam; Interview; Achievements; Clinical experience | 3.0 |
| Theory exam; Interview; Clinical case discussion, English level (oral, listening and reading) | 3.0 |
| Theory exam; Interview; External organisation exams | 3.0 |
| Theory exam; Interview; External organisation exams; Achievements | 3.0 |
| Theory exam; Interview; External organisation exams; Grade point average (GPA) | 3.0 |
| Theory exam; Interview; Grade point average (GPA) | 3.0 |
| Theory exam; Interview; Recommendation /supporting letter | 6.1 |
| Theory exam; Preclinical exam; Clinical exam; Interview; Presentation; Grade point average (GPA); Clinical experience; Academic experience | 3.0 |
| Theory exam; Preclinical exam; Interview | 3.0 |
| Theory exam; Preclinical exam; Interview; Clinical experience; Academic experience | 3.0 |

**Supplementary Table 4: Teaching methods used in postgraduate endodontic program**

| **Options** | **Percentage (%)** |
| --- | --- |
| Face-to-face lectures; Seminars and tutorials; Assignment/Projects; E-learning; Laboratory & practical learning; Clinical cases; Reflective portfolio | 3.0 |
| Face-to-face lectures; Seminars and tutorials; Assignment/Projects; Laboratory & practical learning; Reading lists; Clinical cases; Problem based learning; Chairside teaching | 3.0 |
| Face-to-face lectures; Seminars and tutorials; Assignment/Projects; Reading lists; Clinical cases; Chairside teaching | 3.0 |
| Face-to-face lectures; Seminars and tutorials; Independent study; Assignment/Projects;E-learning; Laboratory & practical learning; Clinical cases; Chairside teaching | 3.0 |
| Face-to-face lectures; Seminars and tutorials; Independent study; Assignment/Projects;E-learning; Laboratory & practical learning; Manuals; Reading lists; Clinical cases; Chairside teaching; Reflective portfolio | 3.0 |
| Face-to-face lectures; Seminars and tutorials; Independent study; Assignment/Projects;E-learning; Laboratory & practical learning; Manuals; Reading lists; Clinical cases; Problem based learning; Chairside teaching | 3.0 |
| Face-to-face lectures; Seminars and tutorials; Independent study; Assignment/Projects;E-learning; Laboratory & practical learning; Video; Manuals; Reading lists; Clinical cases; Problem based learning; Chairside teaching; Reflective portfolio | 6.1 |
| Face-to-face lectures; Seminars and tutorials; Independent study; Assignment/Projects;E-learning; Laboratory & practical learning; Video; Manuals; Reading lists; Clinical cases; Problem based learning; Chairside teaching; Reflective portfolio; clinical accompaniment service | 3.0 |
| Face-to-face lectures; Seminars and tutorials; Independent study; Assignment/Projects; Laboratory & practical learning; Manuals; Reading lists; Clinical cases; Chairside teaching | 3.0 |
| Face-to-face lectures; Seminars and tutorials; Independent study; Assignment/Projects; Laboratory & practical learning; Manuals; Reading lists; Clinical cases; Problem based learning; Chairside teaching; Reflective portfolio | 6.1 |
| Face-to-face lectures; Seminars and tutorials; Independent study; Assignment/Projects; Laboratory & practical learning; Reading lists; Clinical cases; Chairside teaching | 3.0 |
| Face-to-face lectures; Seminars and tutorials; Independent study; Assignment/Projects; Laboratory & practical learning; Reading lists; Clinical cases; Problem based learning; Chairside teaching | 6.1 |
| Face-to-face lectures; Seminars and tutorials; Independent study; Assignment/Projects; Laboratory & practical learning; Video; Manuals; Reading lists; Clinical cases; Chairside teaching | 3.0 |
| Face-to-face lectures; Seminars and tutorials; Independent study; Assignment/Projects; Laboratory & practical learning; Video; Manuals; Reading lists; Clinical cases; Problem based learning; Chairside teaching; Reflective portfolio | 3.0 |
| Face-to-face lectures; Seminars and tutorials; Independent study; Assignment/Projects; Video; Manuals; Reading lists; Clinical cases; Chairside teaching; Reflective portfolio | 3.0 |
| Face-to-face lectures; Seminars and tutorials; Independent study; Laboratory & practical learning; Clinical cases; Chairside teaching | 6.1 |
| Face-to-face lectures; Seminars and tutorials; Independent study; Laboratory & practical learning; Reading lists; Clinical cases | 3.0 |
| Face-to-face lectures; Seminars and tutorials; Laboratory & practical learning; Clinical cases | 6.1 |
| Face-to-face lectures; Seminars and tutorials; Laboratory & practical learning; Clinical cases; Problem based learning | 3.0 |
| Face-to-face lectures; Seminars and tutorials; Reading lists; Clinical cases; Chairside teaching | 3.0 |
| Seminars and tutorials; Assignment/Projects; E-learning; Laboratory & practical learning; Video; Reading lists; Clinical cases; Chairside teaching | 3.0 |
| Seminars and tutorials; Independent study; Assignment/Projects; E-learning; Laboratory & practical learning; Reading lists; Clinical cases; Chairside teaching; Reflective portfolio | 3.0 |
| Seminars and tutorials; Independent study; Assignment/Projects; Laboratory & practical learning; Clinical cases; Chairside teaching; Reflective portfolio | 3.0 |
| Seminars and tutorials; Independent study; Assignment/Projects; Laboratory & practical learning; Manuals; Reading lists; Clinical cases; Chairside teaching; Journal Club and Case Presentations | 3.0 |
| Seminars and tutorials; Independent study; Assignment/Projects; Laboratory & practical learning; Manuals; Reading lists; Clinical cases; Problem based learning; Chairside teaching; Reflective portfolio | 3.0 |
| Seminars and tutorials; Independent study; Assignment/Projects; Reading lists; Clinical cases | 3.0 |
| Seminars and tutorials; Independent study; Laboratory & practical learning; Clinical cases | 3.0 |
| Seminars and tutorials; Independent study; Laboratory & practical learning; Reading lists; Clinical cases; Problem based learning; Chairside teaching; Reflective portfolio | 3.0 |

**Supplementary Table 5: Type of treatments students perform during pre-clinical training**

| **Options** | **Percentage (%)** |
| --- | --- |
| Root canal treatment of multi-rooted teeth; Root canal re-treatment; Removal of fractured instruments | 3.0 |
| Root canal treatment of single-rooted teeth; Root canal treatment of multi-rooted teeth | 12.1 |
| Root canal treatment of single-rooted teeth; Root canal treatment of multi-rooted teeth; Endodontic surgery; Perforation repair; Removal of fractured instruments | 3.0 |
| Root canal treatment of single-rooted teeth; Root canal treatment of multi-rooted teeth; Root canal re-treatment | 6.1 |
| Root canal treatment of single-rooted teeth; Root canal treatment of multi-rooted teeth; Root canal re-treatment; Endodontic surgery; Treatment of teeth with open apices | 3.0 |
| Root canal treatment of single-rooted teeth; Root canal treatment of multi-rooted teeth; Root canal re-treatment; Endodontic surgery; Treatment of teeth with open apices; Management of any accident that occurs during treatment | 3.0 |
| Root canal treatment of single-rooted teeth; Root canal treatment of multi-rooted teeth; Root canal re-treatment; Endodontic surgery; Treatment of teeth with open apices; Perforation repair; Removal of fractured instruments | 6.1 |
| Root canal treatment of single-rooted teeth; Root canal treatment of multi-rooted teeth; Root canal re-treatment; Perforation repair; Removal of fractured instruments | 12.1 |
| Root canal treatment of single-rooted teeth; Root canal treatment of multi-rooted teeth; Root canal re-treatment; Removal of fractured instruments | 9.1 |
| Root canal treatment of single-rooted teeth; Root canal treatment of multi-rooted teeth; Root canal re-treatment; Treatment of teeth with open apices; Perforation repair | 3.0 |
| Root canal treatment of single-rooted teeth; Root canal treatment of multi-rooted teeth; Root canal re-treatment; Treatment of teeth with open apices; Perforation repair; Removal of fractured instruments | 3.0 |
| Vital pulp therapies including pulp capping and pulpotomy; Root canal treatment of multi-rooted teeth; Root canal re-treatment; Endodontic surgery; Treatment of teeth with open apices; Management of traumatic dental injuries; Removal of fractured instruments | 3.0 |
| Vital pulp therapies including pulp capping and pulpotomy; Root canal treatment of single-rooted teeth; Root canal treatment of multi-rooted teeth; Root canal re-treatment; Endodontic surgery; Perforation repair; Removal of fractured instruments | 3.0 |
| Vital pulp therapies including pulp capping and pulpotomy; Root canal treatment of single-rooted teeth; Root canal treatment of multi-rooted teeth; Root canal re-treatment; Endodontic surgery; Treatment of teeth with open apices; Management of traumatic dental injuries; Perforation repair; Management of tooth resorption; Removal of fractured instruments | 6.1 |
| Vital pulp therapies including pulp capping and pulpotomy; Root canal treatment of single-rooted teeth; Root canal treatment of multi-rooted teeth; Root canal re-treatment; Endodontic surgery; Treatment of teeth with open apices; Management of traumatic dental injuries; Perforation repair; Management of tooth resorption; Removal of fractured instruments; Endodontics in teeth with anatomical variations such as dens invaginatus and C-shaped canals | 3.0 |
| Vital pulp therapies including pulp capping and pulpotomy; Root canal treatment of single-rooted teeth; Root canal treatment of multi-rooted teeth; Root canal re-treatment; Management of traumatic dental injuries | 3.0 |
| Vital pulp therapies including pulp capping and pulpotomy; Root canal treatment of single-rooted teeth; Root canal treatment of multi-rooted teeth; Root canal re-treatment; Treatment of teeth with open apices; Perforation repair; Removal of fractured instruments | 3.0 |
| Vital pulp therapies including pulp capping and pulpotomy; Root canal treatment of single-rooted teeth; Root canal treatment of multi-rooted teeth; Treatment of teeth with open apices | 3.0 |
| Others | 12.1 |

**Supplementary Table 6: Stage of treatment ultrasonic instruments used in pre-clinical training**

| **Options** | **Percentage (%)** |
| --- | --- |
| Access cavity preparation/refinement; Irrigant agitation | 3.0 |
| Access cavity preparation/refinement; Irrigant agitation; Retrieval of fractured instrument; Post removal; Space preparation for retrograde filling | 3.0 |
| Access cavity preparation/refinement; Troughing; Irrigant agitation; Retrieval of fractured instrument | 3.0 |
| Access cavity preparation/refinement; Troughing; Irrigant agitation; Retrieval of fractured instrument; Post removal; Space preparation for retrograde filling | 15.2 |
| Access cavity preparation/refinement; Troughing; Irrigant agitation; Retrieval of fractured instrument; Space preparation for retrograde filling | 6.1 |
| Access cavity preparation/refinement; Troughing; Retrieval of fractured instrument | 3.0 |
| Access cavity preparation/refinement; Troughing; Retrieval of fractured instrument; Post removal | 3.0 |
| Irrigant agitation | 3.0 |
| Irrigant agitation; Retrieval of fractured instrument; Post removal | 6.1 |
| Irrigant agitation; Retrieval of fractured instrument; Post removal; Space preparation for retrograde filling | 6.1 |
| No preclinical for postgraduate students | 3.0 |
| not relevant | 9.1 |
| Not used | 15.2 |
| Not used; Irrigant agitation | 3.0 |
| Retrieval of fractured instrument | 3.0 |
| Retrieval of fractured instrument; Post removal | 3.0 |
| Retrieval of fractured instrument; Post removal; Space preparation for retrograde filling | 6.1 |
| Retrieval of fractured instrument; Post removal; Space preparation for retrograde filling; Retreatment: GP removal | 3.0 |
| Troughing; Retrieval of fractured instrument; Post removal | 3.0 |

**Supplementary Table 7: Type of treatments students perform during clinical training**

| **Options** | **Percentage (%)** |
| --- | --- |
| Vital pulp therapies including pulp capping and pulpotomy; Root canal treatment of multi-rooted teeth; Root canal re-treatment; Endodontic surgery; Treatment of teeth with open apices; Management of traumatic dental injuries; Perforation repair; Management of tooth resorption; Removal of fractured instruments; Internal bleaching | 3.0 |
| Vital pulp therapies including pulp capping and pulpotomy; Root canal treatment of single-rooted teeth; Root canal treatment of multi-rooted teeth; Root canal re-treatment | 3.0 |
| Vital pulp therapies including pulp capping and pulpotomy; Root canal treatment of single-rooted teeth; Root canal treatment of multi-rooted teeth; Root canal re-treatment; Endodontic surgery; Treatment of teeth with open apices; Management of traumatic dental injuries; Perforation repair; Management of tooth resorption; Removal of fractured instruments | 15.2 |
| Vital pulp therapies including pulp capping and pulpotomy; Root canal treatment of single-rooted teeth; Root canal treatment of multi-rooted teeth; Root canal re-treatment; Endodontic surgery; Treatment of teeth with open apices; Management of traumatic dental injuries; Perforation repair; Management of tooth resorption; Removal of fractured instruments; Internal bleaching | 54.5 |
| Vital pulp therapies including pulp capping and pulpotomy; Root canal treatment of single-rooted teeth; Root canal treatment of multi-rooted teeth; Root canal re-treatment; Endodontic surgery; Treatment of teeth with open apices; Management of traumatic dental injuries; Perforation repair; Management of tooth resorption; Removal of fractured instruments; Internal bleaching; endo-rest preparation of intraradicular retainers | 3.0 |
| Vital pulp therapies including pulp capping and pulpotomy; Root canal treatment of single-rooted teeth; Root canal treatment of multi-rooted teeth; Root canal re-treatment; Endodontic surgery; Treatment of teeth with open apices; Management of traumatic dental injuries; Perforation repair; Management of tooth resorption; Removal of fractured instruments; Internal bleaching; INTENTIONAL REPLANTATION, AUTOTRASPLANTS | 3.0 |
| Vital pulp therapies including pulp capping and pulpotomy; Root canal treatment of single-rooted teeth; Root canal treatment of multi-rooted teeth; Root canal re-treatment; Endodontic surgery; Treatment of teeth with open apices; Management of traumatic dental injuries; Perforation repair; Management of tooth resorption; Removal of fractured instruments; Internal bleaching; Post-Endodontic Build-ups including provision of endodontic posts, Pre-Endodontic Buildups | 3.0 |
| Vital pulp therapies including pulp capping and pulpotomy; Root canal treatment of single-rooted teeth; Root canal treatment of multi-rooted teeth; Root canal re-treatment; Endodontic surgery; Treatment of teeth with open apices; Management of traumatic dental injuries; Perforation repair; Management of tooth resorption; Removal of fractured instruments; Internal bleaching; surgical repair of external cervical resorption, crown lengthening ,preparation and cementation of crowns and onlays digital impressions , milling of ceramic crowns | 3.0 |
| Vital pulp therapies including pulp capping and pulpotomy; Root canal treatment of single-rooted teeth; Root canal treatment of multi-rooted teeth; Root canal re-treatment; Management of traumatic dental injuries; Perforation repair; Removal of fractured instruments; Internal bleaching | 3.0 |
| Vital pulp therapies including pulp capping and pulpotomy; Root canal treatment of single-rooted teeth; Root canal treatment of multi-rooted teeth; Root canal re-treatment; Treatment of teeth with open apices; Management of traumatic dental injuries; Perforation repair; Management of tooth resorption; Removal of fractured instruments; Internal bleaching | 6.1 |
| Vital pulp therapies including pulp capping and pulpotomy; Root canal treatment of single-rooted teeth; Root canal treatment of multi-rooted teeth; Root canal re-treatment; Treatment of teeth with open apices; Management of traumatic dental injuries; Perforation repair; Removal of fractured instruments; Internal bleaching | 3.0 |

**Supplementary Table 8: Degree of case complexity**

| **Options** | **Percentage (%)** |
| --- | --- |
| Complicated (high difficulty) | 3.0 |
| Moderate (moderate difficulty) | 6.1 |
| Moderate (moderate difficulty); Complicated (high difficulty) | 30.3 |
| Simple (low difficulty); Moderate (moderate difficulty) | 6.1 |
| Simple (low difficulty); Moderate (moderate difficulty); Complicated (high difficulty) | 54.5 |

**Supplementary Table 9: Stage of treatment ultrasonic instruments used in clinical training**

| Options | Percentage (%) |
| --- | --- |
| Access cavity preparation/refinement; Irrigant agitation; Retrieval of fractured instrument; Post removal | 3.0 |
| Access cavity preparation/refinement; Irrigant agitation; Retrieval of fractured instrument; Post removal; Space preparation for retrograde filling | 6.1 |
| Access cavity preparation/refinement; Post removal; Space preparation for retrograde filling; GP removal during retreatment | 3.0 |
| Access cavity preparation/refinement; Retrieval of fractured instrument; Post removal; Space preparation for retrograde filling | 3.0 |
| Access cavity preparation/refinement; Troughing; Irrigant agitation; Retrieval of fractured instrument | 3.0 |
| Access cavity preparation/refinement; Troughing; Irrigant agitation; Retrieval of fractured instrument; Post removal | 3.0 |
| Access cavity preparation/refinement; Troughing; Irrigant agitation; Retrieval of fractured instrument; Post removal; Space preparation for retrograde filling | 60.6 |
| Access cavity preparation/refinement; Troughing; Retrieval of fractured instrument; Post removal; Space preparation for retrograde filling | 3.0 |
| Irrigant agitation; Retrieval of fractured instrument; Post removal | 3.0 |
| Retrieval of fractured instrument; Post removal | 3.0 |
| Troughing; Irrigant agitation; Retrieval of fractured instrument; Post removal; Space preparation for retrograde filling | 3.0 |
| Troughing; Irrigant agitation; Retrieval of fractured instrument; Space preparation for retrograde filling | 3.0 |
| Troughing; Retrieval of fractured instrument; Post removal; Space preparation for retrograde filling | 3.0 |

**Supplementary Table 10: root canal instruments used for canal shaping during clinical training.**

| **Options** | **Percentage (%)** |
| --- | --- |
| Manual stainless instruments; Manual Ni-Ti files; Rotary systems | 12.1 |
| Manual stainless instruments; Manual Ni-Ti files; Rotary systems; Reciprocating systems | 30.3 |
| Manual stainless instruments; Rotary systems | 9.1 |
| Manual stainless instruments; Rotary systems; Reciprocating systems | 36.4 |
| Rotary systems; Reciprocating systems | 12.1 |

**Supplementary Table 11: Interappointment medicament placement technique in clinics**

| **Options** | **Percentage (%)** |
| --- | --- |
| Direct syringe injection | 48.5 |
| Manual files | 9.1 |
| Spiral filler | 42.4 |
